# Diagnostic Yield After Negative Bidirectional Endoscopy for Iron Deficiency Anaemia: A Single-Centre Audit of Downstream Investigations in the UK Two-Week-Wait Pathway

**DOI:** 10.7759/cureus.109740

**Published:** 2026-05-27

**Authors:** Hassan Iqbal

**Affiliations:** 1 Surgery, Airedale General Hospital, Keighley, GBR

**Keywords:** colorectal cancer, colorectal cancer prevention, gastro-intestinal, git endoscopy, iron deficiency anemia (ida), nhs guidelines, two week wait pathway

## Abstract

Background: Iron deficiency anaemia (IDA) accounts for a significant proportion of all gastroenterology referrals. Current British Society of Gastroenterology (BSG) guidance recommends bidirectional endoscopy as first-line investigation and reserves small-bowel or renal-tract investigation for patients with recurrent IDA or inadequate response to iron replacement therapy (IRT) after negative endoscopy. Advanced imaging modalities represent high financial costs to the NHS, but their diagnostic yield in this specific cohort is debated.

Objective: To describe the utilisation and observed diagnostic yield of downstream investigations following negative bidirectional gastrointestinal endoscopy in patients referred via the IDA two-week-wait (2WW) pathway, and to discuss the implications for resource use within this clinical pathway. This audit was exploratory and was not designed as a formal health-economic evaluation.

Methods: We performed a retrospective single-centre audit of all consecutive adult patients investigated on an IDA 2WW pathway during the study period. Demographic data, biochemical confirmation of IDA, completion of baseline investigations, endoscopic findings, cancer detection, and downstream investigations after negative endoscopy were reviewed. Denominators are reported for each analysis; no imputation was performed.

Results: A retrospective audit was conducted on all patients referred via the IDA 2WW pathway to a single UK secondary care centre between January 2024 and March 2025. Eighty-nine patients were included; the mean age was 71.3 (10.1) years, and 46/89 (51.7%) were male. IDA was biochemically confirmed in 89/89 (100%). Oesophagogastroduodenoscopy (OGD) and colonoscopy were performed in 76/89 (85.4%) and 69/89 (77.5%), respectively; complete bidirectional endoscopy was achieved in 66/89 (74.2%). Cancer was identified in 9/89 patients (10.1% (95% CI 4.7 to 18.3)). Overall, endoscopy was negative in 54/89 (60.7% (95% CI 49.7 to 70.9)); among those completing bidirectional endoscopy, 43/66 had negative examinations (65.2% (95% CI 52.4 to 76.5)). In the negative-endoscopy cohort, capsule endoscopy was performed in 1/54 (1.9% (95% CI 0.0 to 9.9)) and CT/MR enterography in 7/54 (13.0% (95% CI 5.4 to 24.9)). Repeat IDA was documented in 28/54 (51.9% (95% CI 37.8 to 65.7)), but an alternative cause was identified on further testing in only 1/54 patients (1.9% (95% CI 0.0 to 9.9)).

Conclusions: In this audit, downstream investigations after negative endoscopy were used infrequently and identified few additional clinically actionable diagnoses. These findings support selective, guideline-concordant follow-up after high-quality negative bidirectional endoscopy, particularly in patients with recurrent or refractory anaemia, but should be interpreted cautiously given the single-centre design, limited follow-up, and low use of advanced imaging.

## Introduction

Iron deficiency anaemia (IDA) is one of the commonest reasons for referral to gastroenterology and endoscopy services. In adult men and postmenopausal women, it is especially important because chronic gastrointestinal (GI) blood loss, malabsorption, and occult malignancy are frequent underlying mechanisms [1-3]. In the UK, IDA contributes substantially to secondary-care activity and NHS expenditure, and contemporary English hospital data show a marked increase in admissions and hospital spells attributable to IDA, underscoring the service impact of investigation pathways [4].

The ageing demographic of the UK population makes this an increasingly important clinical problem. According to the Office for National Statistics, in 2020, there were over 12 million people aged 65 years and above in the UK, representing approximately 18% of the total population [5]. This proportion is projected to increase to 24% by 2043 [6]. Anaemia in this population is not simply a consequence of ageing but a marker of underlying disease, associated with increased frailty, hospitalisation, and mortality [7]. A UK study of elderly patients with IDA found that only one-third of those referred were appropriate for and underwent bidirectional endoscopy, highlighting the complexities of managing this population [8].

Because IDA may be the presenting feature of colorectal, gastric or, less commonly, renal tract malignancy, current UK practice prioritises timely GI investigation. National Institute for Health and Care Excellence (NICE) suspected-cancer guidance places IDA within urgent colorectal cancer referral pathways, while British Society of Gastroenterology (BSG) guidance recommends urgent GI investigation in adults with a new diagnosis of unexplained IDA [1,9]. Bidirectional endoscopy refers to combined upper GI endoscopy (oesophagogastroduodenoscopy, or OGD) and lower GI evaluation, usually colonoscopy, to assess both the upper and lower GI tract for bleeding, malignancy, inflammatory disease, or other clinically relevant causes of IDA. It remains the standard first-line investigation, with CT colonography - an accepted alternative for selected patients in whom colonoscopy is unsuitable or declined [1].

A clinically difficult question arises once bidirectional endoscopy is negative. Small-bowel capsule endoscopy and cross-sectional enterography can identify vascular, inflammatory and neoplastic lesions, but their yield varies markedly according to case-mix, age, symptom profile, and whether patients have recurrent or refractory anaemia [1,10-14]. Meta-analysis has reported pooled capsule endoscopy yields approaching 47%, but these estimates derive from heterogeneous and often highly selected cohorts referred specifically for suspected small-bowel bleeding [11]. More recent data in uncomplicated asymptomatic IDA suggest more modest yields, a low rate of actionable findings, and very limited detection of malignancy [13].

Long-term outcome studies have also suggested that patients with endoscopy-negative IDA often do well, particularly when anaemia resolves with treatment, whereas recurrent or persistent anaemia identifies a higher-risk subgroup in whom repeat conventional endoscopy or selective further investigation may be warranted [15-17]. BSG guidance therefore recommends small-bowel and renal-tract investigation only after high-quality negative bidirectional endoscopy when IDA recurs, or response to iron therapy is inadequate; with capsule endoscopy being preferred when small-bowel evaluation is required [1].

This approach has proven effective in UK practice. A large UK multicentre study of 2,035 patients with IDA without additional GI symptoms found that cancer was diagnosed in 147 patients (7.2%), with luminal GI cancer in 120 (5.9%) [18]. For luminal cancers, the site was colorectal in 103 (5.0% of all patients), gastric in 11 (0.5%), and oesophageal in six (0.3%) [18].

However, a significant proportion of patients - up to 30% in some series - will have a negative bidirectional endoscopy, meaning no clear GI cause for their anaemia is identified [19]. Twenty per cent of elderly patients with IDA have negative upper and lower endoscopy, and two-thirds of these have a lesion in the small bowel [19]. The optimal management of these patients remains a clinical dilemma. Guidelines suggest consideration of further investigations, including capsule endoscopy to visualise the small bowel, or CT/MR enterography [1,20]. The American Society for Gastrointestinal Endoscopy (ASGE) recommends investigating the small bowel in all patients with unexplained IDA after negative standard dual endoscopic evaluations [20].

The present audit was undertaken to assess real-world utilisation of a UK two-week-wait (2WW) IDA pathway and to quantify the observed diagnostic yield of investigations undertaken after negative endoscopy. The analysis was exploratory and primarily descriptive. We also considered whether the observed pattern of investigation raised implications for resource use, while recognising that the study was not designed to provide a formal value-based care or cost-effectiveness assessment.

## Materials and methods

Study design and setting

This study was conducted as a retrospective single-centre audit at Airedale General Hospital, a UK district general hospital. The audit evaluated all consecutive adult patients referred via the IDA 2WW pathway between January 2024 and March 2025. The sample size was therefore determined by the number of eligible referrals during the predefined audit period; no formal sample size calculation was performed because this was a descriptive service audit rather than a hypothesis-powered comparative study. The study design and reporting were informed by the Strengthening the Reporting of Observational Studies in Epidemiology (STROBE) principles [21].

Data sources and data collection

Data were extracted from electronic patient records, endoscopy reporting systems (including procedure reports and histology), laboratory information systems, and outpatient clinic correspondence. Data collection was performed using a structured data extraction template to ensure consistency across cases. Extracted variables included age, sex, biochemical confirmation of IDA, coeliac screening, OGD, colonoscopy, CT colonography, completion of bidirectional endoscopy, endoscopic findings, malignancy detection, post-endoscopy capsule endoscopy, CT/MR enterography, renal tract imaging, recurrent or persistent IDA, and whether a further clinically relevant cause was ultimately identified. IDA was defined using standard laboratory criteria in line with BSG guidance: haemoglobin <130 g/L in men and <120 g/L in women, with ferritin <30 μg/L or transferrin saturation <20% [1].

Inclusion criteria

All adult patients (≥18 years) referred on the IDA 2WW pathway with confirmed IDA were included. Consecutive sampling was used to reduce selection within the local pathway and to reflect routine clinical practice. Patients were excluded if iron deficiency was not biochemically confirmed, if the referral related to anaemia without evidence of iron deficiency, if duplicate referrals occurred during the study period, or if records were insufficient to determine pathway outcome. Patients who did not complete bidirectional endoscopy were retained in the overall pathway analysis, and a separate subgroup analysis was reported for those who completed bidirectional endoscopy before being classified as negative.

Definitions and missing data

Complete bidirectional endoscopy was defined as both OGD and colonoscopy being performed; CT colonography was considered an alternative lower GI investigation when colonoscopy was unsuitable or declined. Negative endoscopy was defined as the absence of a clinically significant GI lesion judged to explain IDA. A clinically actionable downstream finding was defined as a new diagnosis after negative endoscopy that plausibly explained IDA or changed subsequent management. Downstream investigations included capsule endoscopy, CT/MR enterography, and renal tract imaging performed after negative endoscopy. Analyses were undertaken using available data only, with denominators reported for each variable. No statistical imputation was performed. Missing or undocumented investigations were not assumed to have been completed.

Statistical analysis

Analyses were performed in Python (Python Software Foundation, Wilmington, Delaware, USA) using open-source scientific libraries. Continuous data are described as mean (standard deviation) or median (interquartile range), as appropriate. Categorical variables are summarised as counts and percentages, with exact binomial 95% confidence intervals for key proportions. Exploratory comparisons between patients with negative versus non-negative endoscopy used Welch's t-test for age and Fisher's exact test for categorical variables. These comparisons were performed to describe potential group differences and were not intended to support causal inference. A two-sided p-value <0.05 was considered statistically significant. Given the audit design, small sample size, and low event numbers, no multivariable modelling was performed because resulting estimates would be unstable and at risk of overfitting.

## Results

Eighty-nine patients were included. The mean age was 71.3 years (SD 10.1), median age 73 years (IQR 67-78), and 46 (51.7%) were male. IDA was confirmed biochemically in all 89 patients (100%), indicating good case ascertainment for the pathway.

Regarding first-line investigation, OGD was performed in 76 patients (85.4%) and colonoscopy in 69 (77.5%). CT colonography was used as an alternative lower GI investigation in 16 (18.0%). Overall, complete bidirectional endoscopy was achieved in 66 patients (74.2%), as noted in Table 1. While this is lower than rates achieved in some dedicated tertiary centres where same-session bidirectional endoscopy is routinely performed, it reflects real-world practice where patient factors and resource constraints influence completion rates. This finding aligns with a study of elderly patients (≥75 years) with IDA, which found that only 33% of patients initially referred underwent bidirectional endoscopy, with 30.5% either not suitable, choosing not to be investigated, or not attending appointments [8].

**Table 1:** Baseline characteristics and pathway completion IDA: iron deficiency anaemia; OGD: oesophagogastroduodenoscopy; CT: computed tomography

Variable	n/N	Value
Age, mean (SD)	-	71.3 (10.1)
Age, median (IQR), years	-	73 (67-78)
Male sex	46/89	51.7%
IDA confirmed biochemically	89/89	100.0%
Coeliac screen performed	89/89	100.0%
OGD performed	76/89	85.4%
Colonoscopy performed	69/89	77.5%
CT colonography performed	16/89	18.0%
Complete bidirectional endoscopy achieved	66/89	74.2%
Cancer detected	9/89	10.1% (95% CI 4.7 to 18.3)

**Figure 1:**
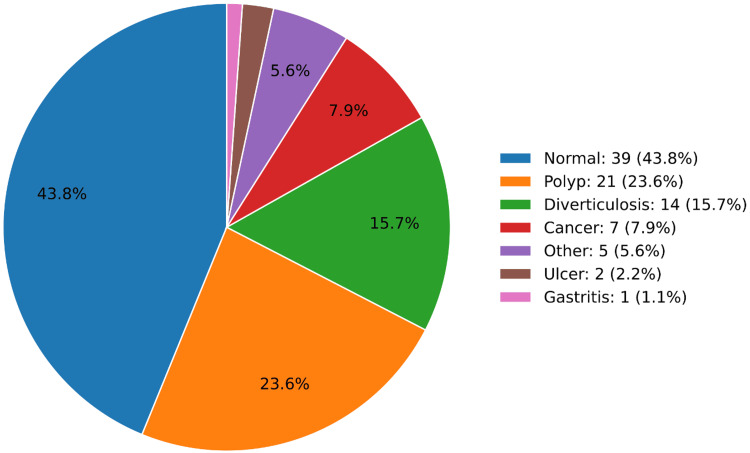
Distribution of index endoscopic findings

Endoscopic findings were diverse, as shown in Figure 1. The commonest documented category was normal examination in 39 cases (43.8%), followed by polyps in 21 (23.6%), diverticulosis in 14 (15.7%), cancer in seven (7.9%), "other" findings in five (5.6%), ulcer disease in two (2.2%), and gastritis in one (1.1%). Cancer was recorded in 9 of 89 patients overall (10.1% (95% CI 4.7 to 18.3)), reflecting an appreciable cancer yield at entry to the urgent pathway. This detection rate exceeds the 7.2% yield reported in the large UK multicentre study of 2,035 patients [18] and the 2% rate found in some UK district general hospital settings [22], while being comparable to international series [23]. The higher rate in our cohort may reflect the selected 2WW population with suspected cancer, compared to the unselected IDA populations.

Malignancies identified included colorectal adenocarcinoma (n=6), caecal tumour (n=1), gastrointestinal stromal tumour (GIST) (n=1), and lung cancer (n=1, diagnosed on subsequent imaging). The detection of lung cancer following normal endoscopy underscores the importance of maintaining a broad differential diagnosis in older patients.

Overall, endoscopy was classified as negative in 54 of 89 patients (60.7% (95% CI 49.7 to 70.9)). When analysis was restricted to those with complete bidirectional endoscopy, 43 of 66 patients had negative examinations (65.2% (95% CI 52.4 to 76.5)). Patients with negative endoscopy were slightly younger than those with a positive finding (mean 70.2 vs 73.0 years), but the difference was not statistically significant (p=0.219). Sex distribution was similar between groups (female 48.1% vs 48.6%; p=1.000).

In the negative-endoscopy cohort, downstream testing was uncommon. Only 1 of 54 patients underwent capsule endoscopy (1.9% (95% CI 0.0 to 9.9)), and seven underwent CT/MR enterography (13.0% (95% CI 5.4 to 24.9)). No renal tract imaging was documented in any case. Repeat IDA was nevertheless recorded in 28 of 54 patients (51.9% (95% CI 37.8 to 65.7)), indicating that recurrent or persistent anaemia after negative endoscopy was not rare.

The observed yield of downstream investigation within this pathway was low. Table 2 shows that only one patient in the negative-endoscopy cohort had a further clinically relevant cause documented (1.9% (95% CI 0.0 to 9.9)), and this was prostate cancer rather than a small-bowel or renal-tract diagnosis. In the stricter subgroup with both negative and complete bidirectional endoscopy, the yield remained 1 of 43 (2.3% (95% CI 0.1 to 12.3)). Among the 20 patients in this subgroup with repeat IDA, a further cause was identified in one patient (5.0%; exact 95% CI 0.1% to 24.9%). These results describe observed pathway outcomes and should not be interpreted as an estimate of the diagnostic performance of capsule endoscopy, which was performed in only one patient.

**Table 2:** Outcomes after negative endoscopy P-values are not shown because the negative complete bidirectional endoscopy group is a nested subgroup of the overall negative-endoscopy cohort and is therefore not an independent comparison group. CI: confidence interval; IDA: iron deficiency anaemia; CT: computed tomography; MR: magnetic resonance

Metric	Overall negative endoscopy, n/N (%)	95% CI	Negative complete bidirectional endoscopy, n/N (%)	95% CI
Negative examinations	54/89 (60.7)	49.7 to 70.9	43/66 (65.2)	52.4 to 76.5
Capsule endoscopy performed	1/54 (1.9)	0.0 to 9.9	1/43 (2.3)	0.1 to 12.3
CT/MR enterography performed	7/54 (13.0)	5.4 to 24.9	7/43 (16.3)	6.8 to 30.7
Renal tract imaging performed	0/54 (0.0)	-	0/43 (0.0)	-
Repeat IDA confirmed	28/54 (51.9)	37.8 to 65.7	20/43 (46.5)	31.2 to 62.3
Further cause identified	1/54 (1.9)	0.0 to 9.9	1/43 (2.3)	0.1 to 12.3

Exploratory comparisons between patients with negative and non-negative endoscopies are summarised in Table 3. Patients with negative endoscopy were slightly younger than those with a positive finding, but this difference was not statistically significant. Sex distribution was similar between groups. OGD completion differed between groups, but this likely reflects pathway completion and case selection rather than a clinically meaningful independent association. The dominant practical message from the audit is that after negative endoscopy, additional investigations were used sparingly and returned very few new diagnoses.

**Table 3:** Exploratory comparisons by endoscopy outcome P-values are exploratory and unadjusted; age was compared using Welch's t-test and categorical variables using Fisher's exact test. OGD: oesophagogastroduodenoscopy

Variable	Negative endoscopy (n=54)	Non-negative endoscopy (n=35)	p-value
Age, mean years	70.2	73.0	0.219
Female sex	26/54 (48.1%)	17/35 (48.6%)	1.000
OGD performed	50/54 (92.6%)	26/35 (74.3%)	0.029

## Discussion

This audit addresses a common but under-reported clinical dilemma: how patients are managed after negative bidirectional endoscopy for IDA within an urgent referral pathway. The principal finding is not that any individual downstream test lacks diagnostic utility, but that further investigations were used infrequently in routine practice and identified few additional clinically actionable diagnoses during the observed period. The result therefore supports the need for selective, guideline-concordant downstream investigation, while recognising that the present audit is underpowered to evaluate the diagnostic performance of individual modalities.

The high yield of initial endoscopy

The 10.1% cancer detection rate in our cohort reinforces the validity of the 2WW IDA referral pathway and compares favourably with published UK rates of 2-7.2% [18,22]. This variation likely reflects differences in referral pathways, population selection, and healthcare settings. The 2WW pathway explicitly selects patients with suspected cancer, which would be expected to yield higher detection rates than unselected IDA populations. Our findings support the continued use of IDA as a red-flag symptom for malignancy and emphasise the importance of prompt, high-quality OGD and colonoscopy as the cornerstone of investigation within the NHS [1].

The majority of actionable diagnoses were made at this stage, consistent with published diagnostic yields for bidirectional endoscopy. This underscores the importance of achieving complete endoscopic evaluation whenever clinically appropriate. The Webb study's identification of risk factors for malignancy - age, male sex, lower haemoglobin, and elevated CRP - may help UK clinicians risk-stratify patients for urgency of investigation [18].

The low yield of advanced small bowel imaging in unselected patients

In this cohort, further investigation with capsule endoscopy or CT/MR enterography after negative bidirectional endoscopy was uncommon. Capsule endoscopy was performed in only one patient, meaning that no reliable conclusion can be drawn about its individual diagnostic yield. The audit is better interpreted as an assessment of pathway utilisation and observed downstream findings, rather than a comparative evaluation of small-bowel imaging modalities.

The discrepancy between our findings and meta-analyses reporting 40-61% diagnostic yields highlights the importance of distinguishing between any abnormality and clinically actionable findings [24]. When analysis is restricted to asymptomatic patients with negative bidirectional endoscopy - precisely the population in whom the decision for further testing arises - the yield of actionable findings falls to 8% [13]. Even when abnormalities are detected, they are predominantly angiodysplasias, which may or may not be the true cause of IDA and may not require intervention [19].

This distinction is important when considering resource utilisation within the NHS. Although advanced small-bowel investigations involve direct procedural costs and reporting time, this study did not include a formal economic evaluation and therefore cannot establish cost-effectiveness. Instead, the data suggest that routine escalation after every negative endoscopic evaluation may require careful justification and should ideally be guided by recurrent or refractory anaemia, symptoms, treatment response, and patient fitness. The 2023 American Gastroenterological Association (AGA) technical review also supports a trial of iron supplementation before routine small-bowel evaluation in uncomplicated asymptomatic patients [7].

The current dataset also fits with the longer-term outcome literature on endoscopy-negative IDA. Soon et al. found that patients whose anaemia resolved after negative bidirectional endoscopy generally had favourable outcomes, whereas recurrent anaemia identified a subgroup at higher risk of subsequent GI pathology and death [15]. Earlier longitudinal studies similarly concluded that prognosis after negative GI evaluation is often good and that repeated investigation should be focused on those with refractory or recurrent anaemia rather than applied indiscriminately [16,17]. In the present audit, recurrent or persistent IDA was relatively common after negative endoscopy, but even within this subgroup, the observed additional diagnostic return was low.

The role of faecal immunochemical testing (FIT) in risk stratification

Emerging evidence supports the use of FIT in triaging patients with IDA within the NHS. A 2024 systematic review and meta-analysis demonstrated that with a threshold of 10 μg Hb/g, FIT has a sensitivity of 90.7% and specificity of 81.0% for colorectal cancer in patients with ID anaemia [25]. Among patients with normal FIT results, the majority can be safely discharged without invasive testing. The negative predictive value approaches 99.6% for colorectal cancers [25].

The BSG has recently issued guidelines on the role of FIT in the endoscopic investigation of IDA (2025), acknowledging that the rapidly emerging evidence base for FIT has resulted in widespread use as a triage tool between primary and secondary care [26]. These guidelines are additive to existing BSG IDA guidelines [1] and aim to provide clarity on the role of FIT in planning investigations [26]. The guidelines recommend that FIT should be used in primary care to guide referral decisions, with a threshold of 10 μg Hb/g identifying patients at highest risk of colorectal pathology who require urgent investigation [26].

FIT was not systematically used in our cohort, which limits applicability to current and evolving IDA pathways where FIT is increasingly incorporated into risk stratification. Future pathway evaluations should include FIT results, anaemia severity, treatment response, and longer-term outcomes to determine how non-invasive testing can refine selection for endoscopy and downstream investigation.

The impact of patient factors

A significant minority (25.8%) of patients did not complete bidirectional endoscopy, primarily due to frailty, comorbidity, or refusal. This is a real-world reality that guideline developers must acknowledge. A study of patients aged 75 years and older found that only 33% of patients initially referred underwent bidirectional endoscopy, with 30.5% either not suitable, choosing not to be investigated, or not attending appointments [8]. The study recommended a one-stop initial clinic assessment in this group of patients rather than a straight-to-test approach, which is likely to result in inefficiencies in endoscopy slots and inappropriate investigations in a high-risk group [8].

In patients where bidirectional endoscopy is not appropriate, options include OGD and CT colonography with faecal tagging, CT scan of abdomen/pelvis, or treatment of anaemia without investigation [8]. The 13.0% rate of CT colonography use in our cohort represents an acceptable alternative when colonoscopy is not feasible or declined.

The study also has service implications. Dedicated IDA services can place a substantial demand on endoscopy capacity; data from a long-running UK IDA clinic suggest that IDA investigation may account for more than one-fifth of diagnostic endoscopies [27]. National English data show rising admissions and costs for IDA care, with markedly higher costs for non-elective than elective management [4]. In that context, a policy of reflex downstream small-bowel imaging after every negative bidirectional investigation would be difficult to justify without strong evidence of additional clinically meaningful yield. Our findings suggest that indiscriminate downstream investigation is unlikely to be efficient in a pathway already achieving good baseline screening and a meaningful upfront cancer detection rate.

The observed cancer detection rate of just over 10% in the whole cohort is consistent with the recognised malignant potential of unexplained IDA in older adults [1,28]. The finding reinforces the value of prompt first-line investigation rather than diminishing it. In other words, the audit argues for maintaining urgency at the front end of the pathway while being more selective at the back end once bidirectional endoscopy is negative.

Study limitations

This study has several limitations. It is a retrospective, single-centre audit with a relatively small sample size (n=89), which limits external validity, statistical power, and the ability to perform robust subgroup or multivariable analyses. Inclusion was restricted to patients referred via the 2WW pathway, introducing selection bias toward a higher-risk population and limiting applicability to unselected IDA populations, primary-care cohorts, and international settings. Complete bidirectional endoscopy was achieved in 74.2% of patients; although a complete-endoscopy subgroup was reported, incomplete investigation limits certainty when interpreting the overall negative-endoscopy cohort. Downstream investigations were used infrequently, particularly capsule endoscopy, so the study cannot estimate the diagnostic performance of individual advanced imaging modalities. Longitudinal follow-up beyond available routine records was not performed; therefore, missed diagnoses, delayed cancers, and true negative outcomes cannot be fully assessed. FIT was not systematically used, limiting relevance to newer pathways incorporating FIT-based risk stratification. Finally, no formal cost-effectiveness analysis was undertaken, and findings relating to resource utilisation should be interpreted as exploratory rather than economic conclusions.

Despite these limitations, the audit has practical value as a real-world description of pathway completion, investigation utilisation, and observed downstream outcomes in a UK district general hospital. It highlights areas for service improvement and future research, including standardised follow-up, systematic FIT use, clearer definitions of clinically actionable findings, and multicentre datasets large enough to evaluate predictors of downstream diagnostic yield.

## Conclusions

This single-centre audit demonstrates that while first-line bidirectional endoscopy remains an essential and effective investigation for IDA, further testing following negative endoscopy provides limited additional diagnostic value in unselected patients. These findings support a selective, patient-centred approach in which further investigations are reserved for those with persistent or recurrent anaemia rather than applied routinely. Real-world constraints, including patient frailty and incomplete pathway adherence, highlight the importance of flexible investigation strategies and shared decision-making. Improved risk stratification, including the use of non-invasive tools, may help optimise resource utilisation, and larger prospective multicentre studies with systematic FIT use, complete follow-up, and formal health-economic evaluation are needed to guide future pathways.
